# The Effect of Diabetes Control Status on CT Findings in Pulmonary Tuberculosis: Emphasis on Bronchial Erosive Changes

**DOI:** 10.3390/jcm12144725

**Published:** 2023-07-17

**Authors:** Min Kyung Jung, Sang Young Lee, Jeong Min Ko, Soo-Ah Im

**Affiliations:** 1 Department of Radiology, St. Vincent’s Hospital, College of Medicine, The Catholic University of Korea, Seoul 06591, Republic of Korea; radmin@catholic.ac.kr (M.K.J.); easayoung91@gmail.com (S.Y.L.); 2 Department of Radiology, Seoul St. Mary’s Hospital, College of Medicine, The Catholic University of Korea, Seoul 06591, Republic of Korea

**Keywords:** tuberculosis, diabetes mellitus, glycemic control, computed tomography

## Abstract

Purpose: Studies on the effect of diabetes mellitus (DM) on the radiologic findings of pulmonary tuberculosis (PTB) have reported inconsistent results. These findings may have been influenced by the glycemic control status of the patients studied. To our knowledge, no recent data have described the effect of the DM control status on CT findings in PTB in terms of medium-sized airway involvement that is visualized as bronchial erosion on CT. The aim of this present study was to determine whether the DM control status influenced radiological manifestations in patients with PTB, with an emphasis on bronchial erosive changes. Methods: We conducted a retrospective single-center study on patients who were newly diagnosed with PTB. A total of 426 consecutive patients with PTB who underwent CT scans at the time of diagnosis from 1 January 2017 to 31 March 2020 were included in this study. The included patients were categorized as having no DM (non-DM), controlled DM, or uncontrolled DM. The patient medical charts, microbiology study results, and pulmonary changes on the CT scans were analyzed. Results: Among 426 patients with PTB who underwent CT scans at the time of diagnosis, 91 were excluded either due to undetermined hemoglobin A1C (HbA1C) levels (n = 25) or concomitant pulmonary diseases (n = 66) that would make the analysis of the pulmonary changes on CT scans difficult. Finally, 335 patients were included in this study (224 men and 111 women; mean age, 59 years; range, 16–95 years). Among the 335 patients, 82 (24.5%) had DM and 52 of those (63.4%) had an uncontrolled status. The frequency of cavitation (43% vs. 23% vs. 79%, *p* < 0.001) and bronchial erosion (44% vs. 30% vs. 73%, *p* < 0.001) was significantly different between the three groups. The uncontrolled DM group showed a high frequency of cavitation and bronchial erosion compared to the non-DM (cavitation, *p* < 0.001 and bronchial erosion, *p* < 0.001) and controlled DM groups (*p* < 0.001 and *p* < 0.001). However, the frequency of cavitation and bronchial erosion in the controlled DM group was not different compared to the non-DM group. Conclusion: The glycemic status (HbA1C ≥ 7.0), not the presence of DM, influenced the radiologic manifestations of PTB, especially in terms of medium-sized bronchial involvement, appearing as bronchial erosive changes and the feeding bronchus sign on chest CT scans. This difference in the uncontrolled DM group was likely to contribute to the higher frequency of cavitation.

## 1. Introduction

Some studies revealed that pulmonary tuberculosis (PTB) patients with diabetes mellitus (DM) had a higher frequency of cavitation than PTB patients without DM [1,2,3,4,5,6,7,8], whereas others did not [9,10,11]. Several studies reported that PTB patients with DM had more lower-lung involvement than PTB patients without DM [3,4,5,7,8,12,13,14,15], but others did not [1,2,6,9,10,16]. Some authors [1,2,3,4,7,8] reported that PTB with DM had more diffuse involvement than PTB without DM, but others did not [9,10]. These inconsistent results on the effect of DM on the radiologic findings of PTB have been reported. Meanwhile, several studies suggested that this difference was influenced by the glycemic control status of patients with DM [7,8,17,18].

The studies [9,10,11] that found no difference in the cavitation of PTB between patients with and without DM were all based on plain radiographs. In the routine reading of a chest CT, cavitation seemed to be more common in PTB patients with uncontrolled DM than non-DM, which is acceptable given the results of previous studies [7,8,17,18]. In addition, it is not uncommon to see the concomitant involvement of a medium-sized airway in PTB patients with uncontrolled DM.

In active PTB, two major types of airway involvement, which can be seen on CT, are endobronchial TB, mainly involving the large airways [19] and centrilobular nodules with tree-in-bud with caseous material fillings of the bronchioles [20]. In addition to these two types, cylindrical bronchiectasis within active inflammation associated with focal erosive or aneurysmal changes is a CT feature that shows the involvement of a medium-sized airway (distal to the segmental bronchi), which is occasionally accompanied by the feeding bronchus sign. The feeding bronchus sign is a CT finding that shows the cavity communicating with the dilated airway [21,22]. Therefore, we hypothesized that a higher frequency of cavitation in PTB with uncontrolled DM may be related to a higher frequency of medium-sized bronchial involvement. However, to our knowledge, no recent data have described the effect of the DM control status on CT findings in PTB in terms of medium-sized airway involvement that is visualized as bronchial erosion on CT. Therefore, the aim of this present study was to determine whether the DM control status influenced radiographic manifestations in patients with PTB, with an emphasis on bronchial erosive changes.

## 2. Methods

### 2.1. Study Design and Patients

We conducted a retrospective single-center study of patients who were newly diagnosed with PTB. The diagnosis of PTB was made on positive results for acid-fast bacilli (AFB) staining or polymerase chain reaction (PCR) tests for *Mycobacterium tuberculosis*, the growth of *M. tuberculosis* from sputum or bronchial washes, and positive pathologic results from lung specimens. A total of 426 consecutive patients with PTB who underwent CT scans at the time of diagnosis from 1 January 2017 to 31 March 2020 were included in this study. The included patients were categorized as having no DM (non-DM), controlled DM, or uncontrolled DM. The diagnosis of DM was confirmed by reviewing the medical records of the individuals. Uncontrolled DM was defined as a plasma hemoglobin A1C (HbA1C) level of ≥7.0%, as recommended by the American Diabetes Association [23]. Patients’ medical charts, microbiology study results, and pulmonary changes on CT scans were analyzed.

### 2.2. Chest CT Protocol

Conventional CT with or without the intravenous administration of contrast medium (90 mL for 65 kg at 2–2.5 mL/s) and high-resolution CT (HRCT) were obtained with three multidetector CT scanners. The decision to perform contrast enhancement was made by the attending clinicians based on their suspicion of malignancy or TB lymphadenitis from the chest radiographs of the patients. The parameters for the SOMATOM Definition Flash scanner (Siemens Healthcare, Forchheim, Germany) included: detector collimation, 128 × 0.6 mm; rotation time, 500 ms; pitch, 1.3 for conventional CT and 1.5 for HRCT; 100-kV tube voltage; and automatic tube current modulation for conventional CT. The parameters for the Discovery CT750 HD scanner and Optima CT660 (General Electric Medical Systems, Milwaukee, WI, USA) included: detector collimation, 64 × 0.625 mm; rotation time, 600 ms for conventional CT and 500 ms for HRCT; pitch, 1.375 for conventional CT and 0.984 for HRCT; 100-kV tube voltage; and automatic tube current modulation for both conventional CT and HRCT. For conventional CT, we analyzed axial images with 1 mm slice thickness at 1 mm intervals and a high-spatial-frequency algorithm. For HRCT, we reviewed axial images with 1 mm slice thickness at 3 mm intervals with a high-spatial-frequency algorithm.

### 2.3. Chest CT Interpretation

We analyzed the CT images obtained before the administration of anti-tuberculous medication, the presence or absence of micronodules and their distribution, tree-in-bud, large opacity (consolidation and macronodule), cavitation, ground glass opacity, bronchovascular bundle thickening, interlobular septal thickening, reverse halo sign, galaxy or cluster sign, endobronchial involvement, pleural effusion, lymphadenopathy, extrathoracic involvement, and bronchial erosion.

Bronchial erosion was defined as the presence of at least one of the following: (a) cylindrical bronchial dilatation with focal erosive or aneurysmal changes, and (b) cavity within the consolidation or nodule communicating with the dilated airway (feeding bronchus sign). Bronchial dilatation was characterized by a broncho-arterial ratio greater than 1. Only bronchial dilation within active lesions representing consolidation or a nodule was defined to rule out pre-existing traction bronchiectasis. Bronchial dilatation outside of the aforementioned lesions was not considered. Changes in the lobar and main bronchi were also not included in this definition of bronchial erosion [21].

All CT scans were retrospectively reviewed by three chest radiologists with 2, 11, and 18 years of experience. Interpretations were determined by a consensus reading among at least two of the three reviewers. All reviewers were blinded to the clinical information throughout this study.

### 2.4. Statistical Analysis

For overall comparisons, the data distribution was first evaluated for normality using the Shapiro–Wilk test. When continuous variables were normally distributed, they were compared among non-DM, controlled DM, and uncontrolled DM patients using analysis of variance (ANOVA). When continuous variables were not normally distributed, they were compared using the Kruskal–Wallis test. Categorical variables were analyzed using the Chi-squared test or Fisher’s exact test, as appropriate. When variables were statistically significant in overall comparisons, pairwise comparisons were performed using the Wilcoxon signed-rank test or the *t*-test for non-normally distributed or normally distributed continuous values, respectively, and the Chi-squared test or Fisher’s exact test for categorical values. In these cases, we applied the Bonferroni correction method to adjust the *p*-value, considering the potential false-positive rate incurred by multiple comparisons (between non-DM and controlled DM; between non-DM and uncontrolled DM; between controlled DM and uncontrolled DM). A *p*-value of < 0.05 (*p* < 0.05/3 = 0.017 in three comparisons after Bonferroni correction) was considered to indicate a statistically significant difference.

### 2.5. Ethics Statement

This study was conducted in accordance with the Declaration of Helsinki. It was approved by the Institutional Review Board of The Catholic University of Korea, St. Vincent’s Hospital (protocol code: VC22RESI0217; date of approval: 20 December 2022). The requirement for informed consent was waived due to the retrospective design of this study.

## 3. Results

### 3.1. Patient Characteristics

**Figure 1:**
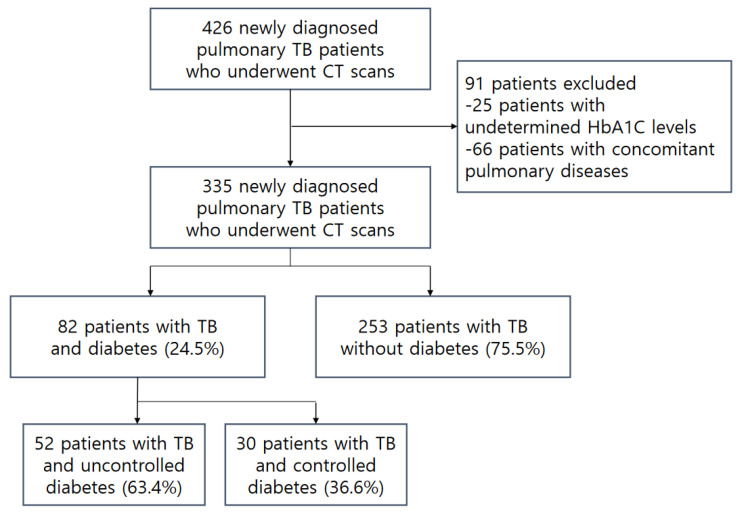
Flow diagram of the study population.

Among the 426 patients with PTB who underwent CT scans at the time of diagnosis, 91 were excluded either due to undetermined HbA1C levels (n = 25) or concomitant pulmonary diseases (pneumonia, n = 22; intrathoracic malignancy and metastasis, n = 8; pulmonary edema, n = 7; severe, old TB, n = 6; lung collapse, n = 5; non-tuberculous mycobacterial infection, n = 4; severe emphysema, n = 7; diffuse interstitial pneumonitis, n = 3; diffuse pulmonary hemorrhage, n = 2; and pneumoconiosis, n = 2) that would make the analysis of the pulmonary changes on CT scans difficult. Finally, 335 patients were included in this study (224 men and 111 women; mean age, 59 years; range, 16–95 years). Among the 335 patients, 82 (24.5%) had DM and 52 of these (63.4%) had an uncontrolled status (Figure 1).

A total of 151 patients had chronic illnesses, including chronic renal disease (n = 9), malignancy (n = 31), human immunodeficiency virus (HIV) infection (n = 1), collagen vascular disease (n = 7), obstructive lung disease (n = 11), and chronic liver disease (n = 29). Some patients had more than one chronic illness.

### 3.2. Clinical Features and Microbiology Results

The clinical characteristics and microbiology results of the patients are summarized in Table 1. The frequency of the positive sputum microbiology results (65% in non-DM vs. 55% in controlled DM vs. 86% in uncontrolled DM, *p* = 0.011), sputum TB-PCR test (46% vs. 40% vs. 80%, *p* < 0.001), sputum AFB smears (34% vs. 23% vs. 61%, *p* = 0.002), and sputum cultures (62% vs. 32% vs. 92%, *p* < 0.001), and the grade of the sputum AFB smear (0.8 ± 1.3 vs. 0.5 ± 1.1 vs. 1.5 ± 1.4, *p* = 0.002) were significantly different between the three groups. The uncontrolled DM group showed a high frequency of positive sputum microbiology results, sputum TB-PCR tests, sputum AFB smears, sputum cultures, and high-grade sputum AFB smears compared to the non-DM (sputum microbiology positivity, *p* = 0.007; sputum TB-PCR positivity, *p* < 0.001; sputum AFB smear positivity, *p* = 0.001; sputum culture positivity, *p* < 0.001; and sputum AFB smear grade, *p* = 0.002) and controlled DM groups (*p* = 0.005, *p* = 0.002, *p* = 0.004, *p* < 0.001, and *p* = 0.005). However, the controlled DM group did not show any difference in the sputum microbiology results compared to the non-DM group.

The AFB smear grades in bronchial washes (0.7 ± 1.1 vs. 0.3 ± 0.7 vs. 1.2 ± 1.4, *p* = 0.008) were significantly different between the three groups. The uncontrolled DM group showed high AFB smear grades in bronchial washes compared to the non-DM (*p* = 0.014) and controlled DM groups (*p* = 0.005). However, the AFB smear grades in bronchial washes in the controlled DM group were not different from those in the non-DM group. The frequency of AFB positivity in bronchial washes (33% vs. 20% vs. 53%, *p* = 0.019) was significantly different between the three groups. The uncontrolled DM group showed a high frequency of AFB positivity in bronchial washes compared to the controlled DM group (*p =* 0.001). But, there was no statistical difference in AFB positivity in bronchial washes between the non-DM and controlled DM group and between the non-DM and uncontrolled DM group.

### 3.3. Chest CT Findings

The frequency of various CT findings in the patients is summarized in Table 2. Peribronchovascular micronodules (76% in non-DM vs. 87% in controlled DM vs. 81% in uncontrolled DM), consolidation/macronodule (87% vs. 83% vs. 92%), and bronchovascular bundle thickening (73% vs. 70% vs. 85%) were the three most common pulmonary parenchymal changes on CT scans in the three groups.

**Figure 2:**
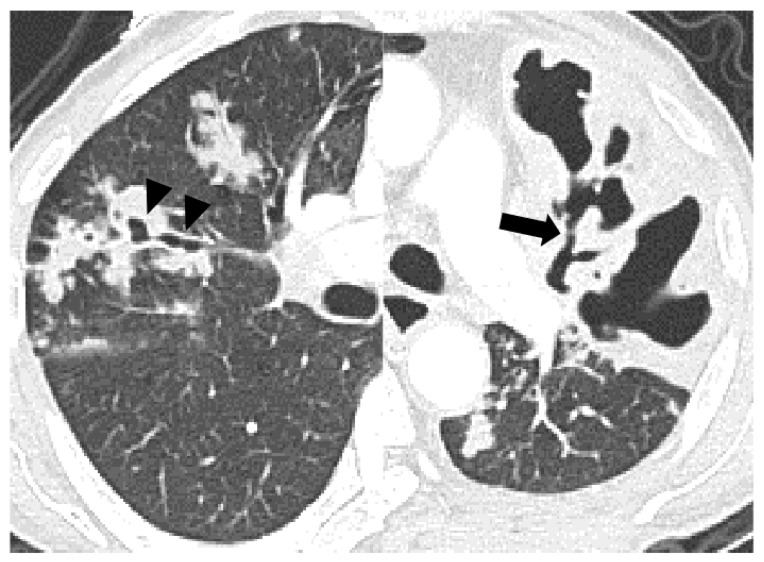
A 67-year-old male with pulmonary tuberculosis and uncontrolled diabetes (HbA1C, 9.90%). The axial CT scan shows fusiform bronchial dilatation with focal erosive or aneurysmal change (arrowheads) within the consolidation in the right upper lobe. The cavity within the consolidation in the left upper lobe communicates with the dilated anterior segmental bronchus (arrow), showing the feeding bronchus sign.

**Figure 3:**
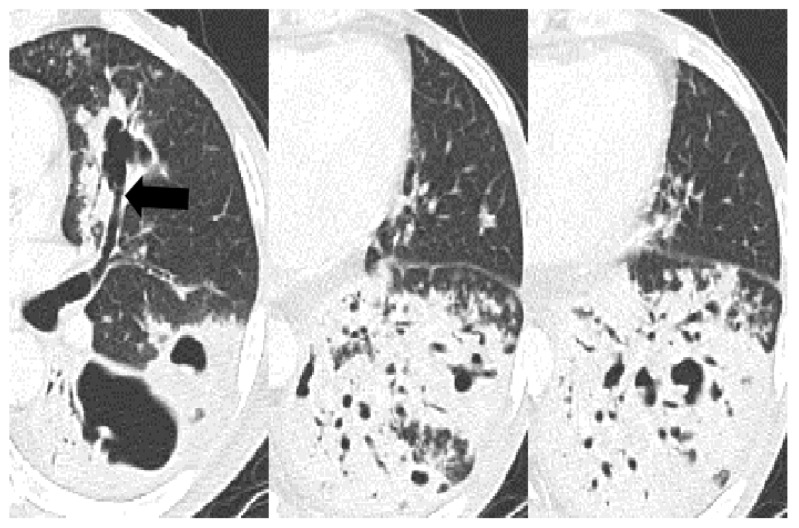
A 55-year-old male with pulmonary tuberculosis and uncontrolled diabetes (HbA1C, 8.30%). Cylindrically dilatated subsegmental bronchus in the superior lingula communicates with the cavity in axial CT scans (feeding bronchus sign, arrow). Multiple feeding bronchus signs and bronchial erosions within the consolidations are also noted in the left lower lobe. Dilated air bronchograms have bead-like nodules hanging from them.

**Figure 4:**
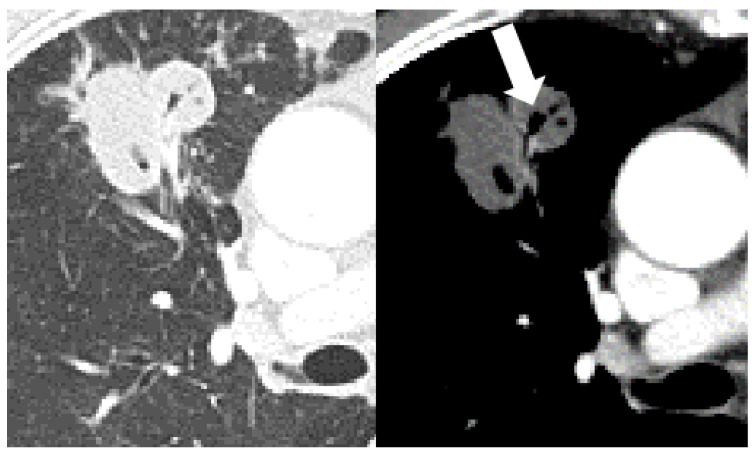
A 70-year-old male with pulmonary tuberculosis and uncontrolled diabetes (HbA1C, 12.3%). The axial CT scan with a lung window setting shows bronchial dilatations within macronodules in the anterior segment of the right upper lobe. Focal bronchial erosion (arrow) is more visible in the mediastinal window setting. Very low-density necrosis of macronodules is also seen.

The frequency of cavitation (43% vs. 23% vs. 79%, *p* < 0.001) and bronchial erosion (44% vs. 30% vs. 73%, *p* < 0.001) were significantly different between the three groups. The uncontrolled DM showed a high frequency of cavitation and bronchial erosion compared to the non-DM (cavitation, *p* < 0.001 and bronchial erosion, *p* < 0.001) and controlled DM groups (*p* < 0.001 and *p* < 0.001). However, the controlled DM group did not show any difference in the frequency of cavitation and bronchial erosion compared to the non-DM group. Other parenchymal changes in the CT scans were not significantly different between the three groups. The cases with distinctive CT findings of PTB in the uncontrolled DM group are seen in Figure 2, Figure 3 and Figure 4.

## 4. Discussion

This study found that differences in the microbiology results and CT findings in patients with PTB were associated with diabetes control status. Patients with uncontrolled DM showed more cavities and more bronchial erosive changes on a chest CT, and higher positive microbiology rates and grades in sputum, than the non-DM and controlled DM groups. However, there were no differences between the controlled DM and non-DM groups.

**Figure 5:**
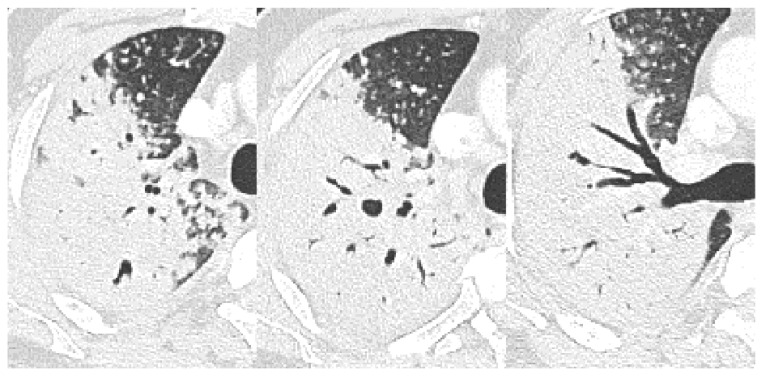
A 64-year-old male with pulmonary tuberculosis and uncontrolled diabetes (HbA1C, 7.2%). Lobar consolidation and surrounding micronodules in the right upper lobe were the main CT findings in this patient. These CT findings could be misdiagnosed as pneumonia, but cylindrical bronchial dilatations with erosive and aneurysmal changes within the consolidation helped to diagnose pulmonary tuberculosis.

Many CT features were more frequently seen in the smear-positive group than in the smear-negative group in patients with active PTB. Among them, cavitation was a significant independent predictive factor of smear-positive sputum results [24]. Also, both the volume and number of cavities were associated with a higher baseline sputum mycobacterial load [25]. Therefore, the high frequency of cavitation and high microbiology positivity rates in the uncontrolled DM group in our study are reasonable. The high frequency of bronchial erosion in the uncontrolled DM group is also likely to be associated with a high positive microbiology rate because it is believed that bronchial wall destruction may occur resulting in bronchial dilatation in PTB [20,22]. Furthermore, about half of the cases with bronchial erosion showed the feeding bronchus sign, which connected the cavity to the dilated airway [21]. This also suggests the existence of bronchial destruction caused by active inflammation. In other words, bronchial dilatations with erosive changes, which are detected within the consolidation, help to differentiate PTB from necrotizing pneumonia (Figure 5). Bronchial dilatation with focal erosion caused by inflammation and the destruction of the bronchial wall has not been reported until now in other necrotizing pneumonia, except in non-tuberculous mycobacterium infections [26].

The results regarding the effect of DM on the radiologic findings of PTB vary substantially between studies. Several studies reported that PTB patients with DM had more lower-lung involvement than PTB patients without DM [3,4,5,7,8,12,13,14,15], but others did not [1,2,6,9,10,16]. Some studies found that PTB patients with DM had a higher frequency of cavitation than PTB patients without DM [1,2,3,4,5,6,7,8], whereas others did not [9,10,11]. Some authors [1,2,3,4,7,8] reported that PTB with DM had more diffuse involvement than PTB without DM, but others did not [9,10]. These differences, according to our data, may be attributed to variances in research populations and, specifically, the number of patients with controlled DM included in the study cohort. In addition, the studies that found no difference in the cavitation of PTB between patients with and without DM were all based on plain radiographs.

Several studies suggested that radiologic manifestations were influenced by the glycemic control status of DM [7,8,17,18,27]. In one study [17], there were no differences in the chest X-ray findings and sputum AFB smear results between patients with controlled DM and non-DM. However, patients with uncontrolled DM (HbA1C ≥ 7.0%) had more cavitary lesions on chest X-rays and higher positive smear rates compared to those without DM. Our study also found that the controlled DM and non-DM groups exhibited similarities in microbiology positivity and the frequency of cavities and bronchial erosions on CT scans. However, patients with uncontrolled DM showed differences in the microbiology results and CT features compared to the non-DM and controlled DM groups. To our knowledge, there has been only one study [8] to date that has investigated whether diabetes control status affects CT findings in PTB patients. In that study, the CT findings that were significantly more common in the HbA1c > 8% diabetes group were lymphadenopathy, more than one cavity in any single lesion, and all lobes involvement. They did not analyze bronchial erosion on the chest CT scans. Considering the high prevalence of bronchial erosion in the uncontrolled DM group in our study, we do not think that bronchial erosive or aneurysmal changes were new radiologic findings. Instead, this lesion was previously neglected or considered non-specific in previous studies. However, our results suggest that bronchial erosive or aneurysmal changes, together with cavities, may be key CT findings in PTB with uncontrolled DM. In summary, our study reinforces that diabetes control status, not the presence or absence of diabetes, influences the radiologic presentation.

In the clinical aspects, uncontrolled DM was a significant risk factor for a positive sputum culture after two months of treatment [17]. Yoon et al. [28] reported that DM itself was not a risk factor for poor treatment response in PTB, but an HbA1C of ≥7% was an independent risk factor in their multicenter prospective cohort study.

Differences in the cytokine response are a possible hypothesis. An animal study [29] found that acute diabetic and euglycemic mice had comparable bacterial burdens in the lungs. In contrast, chronic diabetic mice showed a higher bacterial burden in the lungs than euglycemic mice. Furthermore, there was a decrease in early interferon (IFN)-γ production in the lungs of chronic diabetes mice. The findings of this animal study revealed that chronic hyperglycemia was an offensive condition in the course of PTB. A human study [30] showed that *M. tuberculosis*-specific IFN-γ was inversely associated with fasting blood glucose levels. Therefore, rather than the presence or absence of DM, the control status of diabetes may have an impact on the disease presentation of PTB.

Two major types of airway involvement can be seen in CT findings in active PTB. The first type is endobronchial TB, which mostly affects large airways and is characterized by luminal narrowing and wall thickening in the lobar and main bronchi, usually spreading into the carina and trachea [19]. The second type is centrilobular nodules with tree-in-bud with caseous material fillings of the bronchioles [20]. In addition to these two types, cylindrical bronchiectasis within active lesions (consolidation and nodule) and focal bronchial erosive or aneurysmal changes are CT features that show the involvement of the medium-sized airway and are occasionally accompanied by the feeding bronchus sign. The feeding bronchus sign is a CT finding that shows the cavity communicating with the dilated airway [21,22]. Therefore, we suggest that the higher frequency of cavitation in PTB in patients with uncontrolled DM reported in several studies may be related to a higher frequency of medium-sized bronchial involvement. The control status of DM affects CT manifestations in patients with PTB, starting with bronchial erosive changes and leading to cavitation.

Co-morbidities, such as HIV infection, can affect the disease status and radiological findings of TB, so we performed statistical analysis to see if there was a difference in co-morbidities between the three groups. The co-morbidities analyzed included all chronic diseases that were thought to be closely related to PTB. There was no statistically significant difference in the co-morbidities between the three groups. Therefore, it is unlikely that comorbidities acted as a confounding factor in the results of our study.

This study had limitations that must be acknowledged. The retrospective nature and limited patient sample size in our study may have resulted in selection bias, potentially limiting our conclusions. Further research with larger study populations is required to validate our findings.

## 5. Conclusions

The glycemic status (HbA1C ≥ 7.0%), not the presence of DM, influenced the radiologic manifestations of PTB, especially in terms of medium-sized bronchial involvement, appearing as bronchial erosive changes and the feeding bronchus sign on chest CT scans. This difference in the uncontrolled DM group likely contributed to the higher frequency of cavitation.

## Figures and Tables

**Table 1:** Clinical features, microbiology results, and statistical differences between the three groups.

	Total	Non-DM	Controlled DM	Uncontrolled DM	*p*-Value (1)	*p*-Value (2)	*p*-Value (3)	*p*-Value (4)
	n = 335	n = 253	n = 30	n = 52				
Age, years median (IQR)	59 (47, 73)	57 (41, 72)	71 (63, 81) **	60 (51.5, 68) **	<0.001	<0.001	0.127	<0.001 ‡
Sex, male	224 (67)	158 (63)	21 (70)	45 (87)	0.003	0.417	0.001	0.069
Co-morbidity								
CRF	9 (3)	5 (2)	-	4 (8)	0.086 †	>0.999 †	0.049 †	0.291 †
Malignancy	31 (9)	28 (11)	2 (7)	1 (2)	0.089 †	0.753 †	0.039 †	0.551 †
HIV infection	1 (0)	1 (0)	-	-	>0.999 †	>0.999 †	>0.999 †	-
CVD	7 (2)	5 (2)	2 (7)	-	0.112 †	0.163 †	0.593 †	0.131 †
COPD, asthma	11 (3)	8 (3)	1 (3)	2 (4)	0.875 †	>0.999 †	0.681 †	>0.999 †
Chronic alcoholics, LC	29 (9)	23 (9)	3 (10)	3 (6)	0.749 †	0.746 †	0.590 †	0.664 †
Sputum								
Positive microbiology	178/263 (68)	129/198 (65)	12/22 (55)	37/43 (86)	0.011	0.325	0.007	0.005
Positive TB-PCR test	117/226 (52)	77/166 (46)	8/20 (40)	32/40 (80)	<0.001	0.588	<0.001	0.002
Positive AFB smear	96/256 (38)	65/191 (34)	5/22 (23)	26/43 (61)	0.002	0.285	0.001	0.004
AFB smear grade, mean ± SD	0.9 ± 1.3	0.8 ± 1.3	0.5 ± 1.1	1.5 ± 1.4	0.002	0.238	0.002	0.005
Positive culture	154/239 (64)	113/182 (62)	6/19 (32)	35/38 (92)	<0.001	0.01	<0.001	<0.001
Bronchial washing fluid								
Positive microbiology	204/221 (92)	142/158 (90)	24/25 (96)	38/38 (100)	0.076 †	0.475 †	0.045 †	0.397 †
Positive TB-PCR test	169/219 (77)	116/156 (74)	19/25 (76)	34/38 (90)	0.136	0.861	0.046	0.176 †
Positive AFB smear	76/218 (35)	51/155 (33)	5/25 (20)	20/38 (53)	0.019	0.196	0.024	0.001
AFB smear grade, mean ± SD	0.7 ± 1.1	0.7 ± 1.1	0.3 ± 0.7	1.2 ± 1.4	0.008	0.144	0.014	0.005
Positive culture	191/220 (87)	136/157 (87)	19/25 (76)	36/38 (95)	0.098	0.220 †	0.261 †	0.0498 †

Note: ** Shapiro–Wilk > 0.05. Values are numbers (percentages) for categorical variables unless otherwise indicated. *p*-values (1) were calculated using the Chi-squared test or Fisher’s exact test† for categorical variables and the Kruskal–Wallis test or ANOVA test* for continuous variables (statistically significant at *p* < 0.05). *p*-values (2), (3), and (4) were calculated using the Chi-squared test or Fisher’s exact test† for categorical values and the Wilcoxon signed-rank test or *t*-test‡ for continuous values (statistically significant at adjusted/corrected *p* < 0.017). *p*-value (1): between non-DM and uncontrolled DM and controlled DM; *p*-value (2): between non-DM and controlled DM; *p*-value (3): between non-DM and uncontrolled DM; and *p*-value (4): between controlled DM and uncontrolled DM; TB-PCR, polymerase chain reaction test for *Mycobacterium tuberculosis*; AFB, acid-fast bacilli; CRF, chronic renal failure; HIV, human immunodeficiency virus; CVD, collagen vascular disease; COPD, chronic obstructive pulmonary disease; and LC, liver cirrhosis.

**Table 2:** Frequency of CT findings and statistical differences between the three groups.

	Total	Non-DM	Controlled DM	Uncontrolled DM	*p*-Value (1)	*p*-Value (2)	*p*-Value (3)	*p*-Value (4)
	n = 335	n = 253	n = 30	n = 52				
Micronodule								
Centrilobular	168 (50)	125 (49)	14 (47)	29 (56)	0.651	0.777	0.403	0.427
Peribronchovascular	261 (78)	193 (76)	26 (87)	42 (81)	0.373	0.199	0.484	0.494
Septal	171 (51)	123 (49)	17 (57)	31 (60)	0.286	0.404	0.149	0.794
Subpleural	113 (34)	94 (37)	6 (20)	13 (25)	0.06	0.063	0.094	0.605
Miliary	15 (5)	8 (3)	3 (10)	4 (8)	0.066 †	0.099 †	0.129 †	0.703 †
Tree-in-bud	134 (40)	98 (39)	12 (40)	24 (46)	0.610 †	0.893	0.32	0.589
Consolidation/macronodule	294 (89)	221 (87)	25 (83)	48 (92)	0.452	0.566 †	0.313	0.276 †
Cavitation	157 (47)	109 (43)	7 (23)	41 (79)	<0.001	0.038	<0.001	<0.001
Ground glass opacity	52 (16)	35 (14)	5 (17)	12 (23)	0.241	0.590 †	0.093	0.49
Bronchovascular bundle thickening	249 (74)	184 (73)	21 (70)	44 (85)	0.172	0.752	0.072	0.116
Interlobular septal thickening	235 (70)	176 (70)	20 (67)	39 (75)	0.671	0.745	0.434	0.419
Reverse halo sign	9 (3)	6 (2)	1 (3)	2 (4)	0.587 †	0.548 †	0.628 †	>0.999 †
Galaxy/cluster sign	24 (7)	19 (8)	2 (7)	3 (6)	>0.999 †	>0.999 †	>0.999 †	>0.999 †
Endobronchial involvement	64 (19)	51 (20)	6 (20)	7 (14)	0.53	0.984	0.262	0.534 †
Pleurisy	108 (32)	88 (35)	6 (20)	14 (27)	0.176	0.104	0.274	0.482
Lymphadenopathy	110 (33)	86 (34)	11 (37)	13 (25)	0.406	0.77	0.207	0.263
Extrathoracic involvement	19 (6)	16 (6)	1 (3)	2 (4)	0.847 †	>0.999 †	0.748 †	>0.999 †
Bronchial erosion	157 (47)	110 (44)	9 (30)	38 (73)	<0.001	0.157	<0.001	<0.001

Note: Values are numbers (percentages) for categorical variables unless otherwise indicated. *p*-values (1) were calculated using the Chi-squared test or Fisher’s exact test† for categorical variables and the Kruskal–Wallis test or ANOVA test* for continuous variables (statistically significant at *p* < 0.05). *p*-values (2), (3), and (4) were calculated using the Chi-squared test or Fisher’s exact test† for categorical values and the Wilcoxon signed-rank test or *t*-test‡ for continuous values (statistically significant at adjusted/corrected *p* < 0.017). *p*-value (1): between non-DM and uncontrolled DM and controlled DM; *p*-value (2): between non-DM and controlled DM; *p*-value (3): between non-DM and uncontrolled DM; and *p*-value (4): between controlled DM and uncontrolled DM.

## Data Availability

Data available on request due to restrictions. The data presented in this study are available on request from the corresponding author.
